# Tuning the Functional Properties of Bitter Vetch (*Vicia ervilia*) Protein Films Grafted with Spermidine

**DOI:** 10.3390/ijms18122658

**Published:** 2017-12-08

**Authors:** Raffaele Porta, Prospero Di Pierro, Valentina Roviello, Mohammed Sabbah

**Affiliations:** 1 Department of Chemical Sciences, University of Naples “Federico II”, 80126 Naples, Italy; raffaele.porta@unina.it (R.P.); mohammed.sabbah@unina.it (M.S.); 2 CeSMA, University of Naples “Federico II”, 80126 Naples, Italy; valentina.roviello@unina.it; 3 Department of Nutrition and Food Technology, An-Najah National University, P.O. Box 7 Nablus, Palestine

**Keywords:** edible film, food coating, plasticizer, spermidine, glycerol, *Vicia ervilia*

## Abstract

Bitter vetch protein films containing positively charged spermidine, alone or with low amounts of glycerol, showed high tensile strength that progressively decreased by increasing the plasticizer concentration. Accordingly, lower film elongation at break and higher Young’s module values were detected in the presence of the polyamine without or with small amounts of glycerol. These data suggest that spermidine not only acts as a plasticizer itself by ionically interacting with proteins, but that it also facilitates glycerol-dependent reduction of the intermolecular forces along the protein chains, consequently improving the film flexibility and extensibility. Thus, spermidine may be considered not only as a primary, but also as a secondary plasticizer because of its ability to enhance glycerol plasticizing performance. Such double behavior of the polyamine was confirmed by the film permeability tests, since spermidine increased the barrier properties to gases and water vapor, while glycerol emphasized this effect at low concentrations but led to its marked reversal at high concentrations. Film microscopic images also substantiated these findings, showing more compact, cohesive, and homogeneous matrices in all spermidine-containing films.

## 1. Introduction

Substantial advances have been made over the last two decades in the field of biodegradable polymers, mostly derived from renewable natural resources, to produce bioplastics with features similar to those typical of oil-based materials [1]. In particular, protein-based edible films and coatings have attracted an increasing interest in recent years since they might be used to protect pharmaceuticals or improve the shelf life of different food products [2]. These biomaterials are generally first evaluated for their mechanical and barrier properties as a function of different types and concentrations of plasticizers, generally small and nonvolatile organic additives used to increase film extensibility and reduce its cristallinity, brittleness, and water vapor permeability. Plasticizers normally act by decreasing the intermolecular forces along the polymer chains, thus reducing the relative number of polymer–polymer contacts and producing a decrease in the cohesion and tensile strength, thereby increasing the flexibility of the film by allowing its deformation without rupture [3,4,5]. Consequently, plasticizers generally improve the processability of the different biomaterials. Therefore, as the bioplastic industry is continuously growing, the demand for new kinds of plasticizers endowed with specific characteristics and performances compatible with each single bioplastic is growing in parallel [4,5].

In the attempt to refine the mechanical and barrier properties of hydrocolloid biopolymers, the effect of the aliphatic polyamines spermidine (SPD) and spermine on both polysaccharide- and protein-based films was recently investigated [6,7]. Polyamines are low-molecular-weight polycations, widely distributed in nature and able to mimic the action of divalent ions like Ca^2+^ both in vitro and in vivo [8]. Although the role of polyamines has been associated with aging, various metabolic disorders, cell growth, and cancer, their precise biochemical function is one of the remaining mysteries of molecular cell biology [9]. The very low toxicity of SPD, attested by an acute oral toxicity of 600 mg/kg in rats [10] and by an LD50 value higher than 2000 mg/kg in mice [11], allows its possible addition to edible film-forming solutions (FFSs) to obtain safe food coatings. Thus, SPD effect was tested by including the polyamine in FFSs containing a protein concentrate obtained from bitter vetch (*Vicia ervilia*; BV) seeds and different amounts of glycerol (GLY), added as plasticizer [7]. BV, an annual legume species of the *Vicia* genus, is widely cultivated only for livestock feed in temperate regions of Europe, western and central Asia, north Africa, and Americas [12,13,14,15]. BV seeds, containing up to 25% of protein, were demonstrated to be a potentially useful source for food packaging applications, being not only an abundant protein source but also an inexpensive one for edible film production [15,16,17,18]. It was recently observed [7] that films made with a BV protein concentrate (BVPC) in the absence of high concentrations of GLY are brittle, difficult to manipulate, and, consequently, impossible to study. Moreover, the addition of high amounts of GLY were shown unable to give rise to handleable films even when BV proteins were previously denatured by heat treatment at 80 °C for 30 min. Conversely, the presence of appropriate SPD amounts allowed to obtain easily manipulable films at low GLY concentrations and even in the absence of GLY. Therefore, a plasticizing-like effect and a facilitating action on GLY plasticizing effect has been hypothesized for this aliphatic polycation [7]. In this paper, we report SPD influence on the morphological, mechanical, and barrier properties of films prepared from both native and heat-denatured BV proteins at different concentrations of GLY and pH values.

## 2. Results and Discussion

Since the addition of millimolar concentrations of SPD to BVPC FFSs allows the formation of handleable films even in the absence of GLY [7], we were first stimulated to determine the mechanical properties of these GLY-unplasticized films, as well of those prepared in the presence of different amounts of SPD (2–5 mM, corresponding to about 4–9% *w*/*w* BV protein) and increasing GLY concentrations (4–42 mM GLY, corresponding to about 5–50% *w*/*w* BV protein). To investigate the influence of SPD and GLY on both native and denatured protein-based films, a BVPC pretreated for 30 min at either 25 or 80 °C was used as a film protein source. Furthermore, to study the influence of the positively charged amino groups of SPD in structuring the film protein network and the consequent film mechanical properties, tensile strength (TS), elongation at break (EB), and Young’s module (YM) values detected for films prepared at pH 8.0 (i.e., under SPD amino group p*K*a values) were compared to those detected for films obtained at pH 11.0 (i.e., over SPD amino group p*K*a values) [19].

The data reported in Table 1 indicate that all films made at pH 8.0 with both native and heat-denatured BV proteins and containing SPD alone or with low amounts of GLY showed higher TS, with a maximum value of 12.00 ± 1.21 MPa observed when 3 mM SPD was used in the heat-denatured BVPC FFSs. In addition, the film TS appeared to be progressively lower in all samples containing SPD and increasing GLY concentrations, reaching a very low value (less than 1.0 MPa) at 42 mM GLY. It is worth noting that these latter values are significantly lower than those detected by analyzing films prepared in the presence of GLY alone at high concentrations, thus confirming a positive influence of SPD on GLY plasticizing action.

Consequential and predictable effects were recorded when the EB of the same films was measured (Table 2). In fact, the lowest EB values were detected when analyzing the films prepared from both native and heat-denatured BVPC in the presence of SPD without or with low concentrations of GLY. Also in this case, EB was observed to rise in parallel with the increase of GLY amount contained in the FFSs where SPD was also present, reaching the maximum values (EB > 80%) at the highest GLY concentration (42 mM). Also such values resulted significantly different (much higher) than those detected when the films were prepared in the presence of high GLY concentrations (33 and 42 mM) but in the absence of SPD. 

The same behavior was recorded when measuring the YM of these films, with very high YM values detected for films containing SPD alone (more than 400 MPa) or with low GLY amounts. Also, the YM of SPD-containing films was observed to progressively decrease to very low values (less than 10 MPa) with increasing GLY concentrations up to 42 mM (Table 3). Also in this case, the YM of films containing both SPD and high amounts of GLY resulted much lower than the YM values observed for films prepared in the presence of high concentrations of GLY but in the absence of SPD.

**Figure 1:**
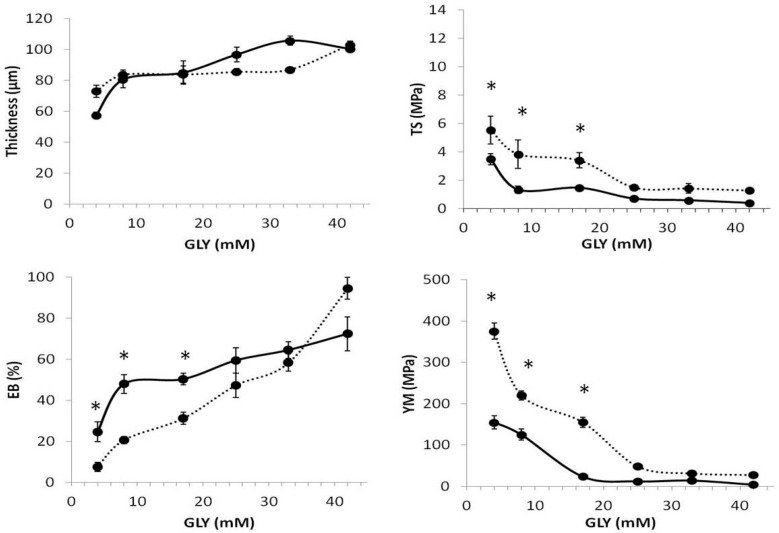
Effect of different GLY concentrations on the mechanical properties of films prepared with BV proteins treated at 25 °C, in the presence of 3 mM SPD at either pH 8.0 (solid line) or pH 11.0 (dotted line). Brittle and unhandleable films were obtained by casting FFSs prepared in the absence of GLY. The results are expressed as mean ± standard deviation (* values significantly different at *p* < 0.05). Further experimental details are given in the text.

**Figure 2:**
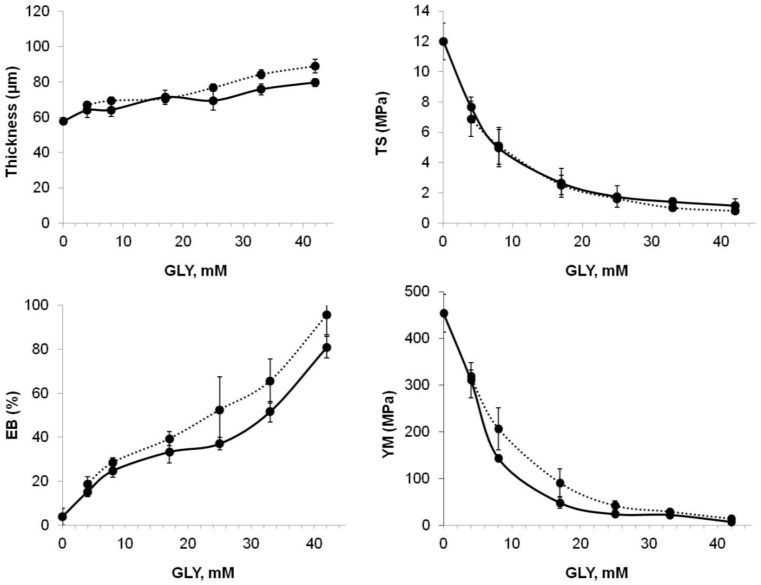
Effect of different GLY concentrations on the mechanical properties of films prepared by BV proteins treated at 80 °C, in the presence of 3 mM SPD at either pH 8.0 (solid line) or pH 11.0 (dotted line). Brittle and unhandleable films were obtained by casting FFSs prepared in the absence of GLY at pH 11.0. The results are expressed as mean ± standard deviation. Further experimental details are given in the text.

All these data support our hypothesis on the ability of SPD not only to act as a plasticizer itself by ionically interacting at pH 8.0 with the negative charges occurring onto BV proteins, but also to facilitate in this way GLY action in reducing the intermolecular forces along the protein chains, and consequently to further improve film flexibility and extensibility. This assumption was confirmed by the data of the mechanical properties of the films prepared in the presence of SPD at pH 11.0 (i.e., when its amino groups are uncharged being over their respective p*K*a values) in comparison with those obtained for the films prepared at pH 8.0 (i.e., when SPD is fully protonated). In fact, Figure 1 clearly indicates that films derived from native BV proteins have different TS, EB, and YM when they are prepared at pH 8.0 or 11.0 in the presence of 3 mM SPD and low GLY concentrations. In particular, the films obtained at pH 8.0 exhibited lower TS and YM and higher EB compared to those prepared at pH 11.0, thus indicating that the different kind of interaction between the polyamine and the folded protein chains is responsible for the different effects on the film mechanical properties. These differences have been proved not to be significant when heat-denatured BV protein films were tested, probably because the unfolded biopolymer chains are influenced by the uncharged SPD in the same way as by the protonated polyamine (Figure 2). In this case, polyamine–protein hydrogen bonds and hydrophobic interactions may be hypothesized. 

Lastly, a further demonstration that SPD was able to enhance the plasticizing performance of GLY was given by the significantly lower EB values (52.56 ± 3.90 vs. 94.45 ± 5.26 for native BV protein films; 51.17 ± 4.80 vs. 95.56 ± 7.62 for denatured BV protein films) and higher YM values (34.11 ± 1.66 vs. 27.66 ± 1.16 for native BV protein films; 68.78 ± 3.60 vs. 13.70 ± 2.70 for denatured BV protein films) detected for films prepared at pH 11.0 in the presence of 42 mM GLY and in the absence of SPD, compared to those containing 42 mM GLY and 3 mM SPD. 

Since a primary plasticizer is generally defined as a molecule that, when added to a material, makes it softer, more flexible, and easier to be processed, our findings lead to consider SPD as a possible primary plasticizer of protein-based films. In fact, the addition of millimolar concentrations of SPD allowed BV proteins to give rise to handleable materials as a result of an increase of their elongation and softeness. However, SPD can also be considered as a secondary plasticizer, namely, an “extender”, because of its ability to enhance the plasticizing performance even of a well-known primary plasticizer such as glycerol [20]. Such double behavior of the polyamine as both a primary and a secondary plasticizer seems to be confirmed also by the analysis of the permeability properties of BVPC films obtained in the presence of different concentrations of SPD and GLY. In fact, the data reported in Table 4 and Table 5 showed that the addition of increasing SPD concentrations into FFSs caused in the derived films an increase in the barrier properties to gases (CO_2_ and O_2_) as well as to water vapor (WV) and that the concomitant presence of low GLY concentrations emphasized this effect. Further increases of GLY concentration into the films, however, led to a marked reversal of the positive barrier effect, thus indicating that an excessive film plasticization promoted film permeability to both gases and WV. It is worthy to note that the same effect was observed by testing films prepared with both native and heat-denatured BV proteins.

**Figure 3:**
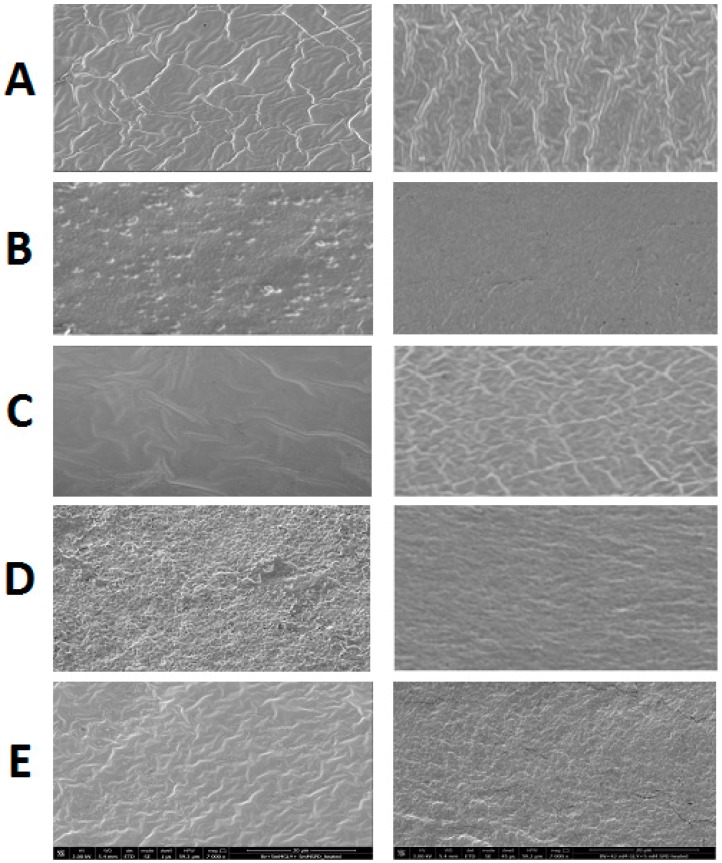
SEM micrographs at 7000× magnification of the film surfaces (left) and cross sections (right) obtained from native (**A**–**C**) and heat-denatured (**D**,**E**) BVPC in the presence of 5 mM SPD with (**C**,**E**) or without (**B**,**D**) 42 mM GLY, as well as in the absence of both SPD and GLY (**A**). The sample prepared with heat-denatured BVPC in the absence of SPD was not analyzed because not handleable for its stickiness. The images shown were chosen as the most representative of each sample. Further experimental details are given in the text.

Figure 3 shows the surface and cross-sectional morphology of films derived from FFSs containing native (A–C) or heat denatured BVPC (D,E), mixed with either GLY (A), SPD alone (B,D), or with both plasticizers (C,E). According to the SEM images, BVPC films containing GLY and SPD together presented a relatively smoother and more uniform and continuous appearance with respect to films containing only GLY, which appeared less cohesive and exhibited an evident heterogeneity. Moreover, also the surfaces and the cross sections of the SPD-containing films prepared in the absence of GLY showed features typical of more compact matrices, likely accountable for the lower permeability detected with all films containing the polyamine. These results are in agreement with the marked reduction of thickness of SPD containing films, prepared with both native and denatured BVPC (Table 6).

Finally, as far as a possible application of SPD/GLY-plasticized films is concerned, we report in Table 7 the mechanical and barrier properties of various hydrocolloid (polysaccharide- and protein-based) films previously described in the literature, as well as those of some commercial bioplastic (Viscofan NDX and Mater-Bi S-301) and plastic (HD-PE 02) materials analyzed in the present study. The comparison of the features of the SPD-containing BVPC films described here with those of the other hydrocolloid materials indicated similar TS values when BVPC films were prepared in the absence of GLY, whereas higher EB characterized the BVPC films when also GLY was present. Conversely, the barrier effects toward both gases and WV seemed to be similar in all films. More in particular, it is worthy to note that the BVPC films prepared in the presence of low concentrations of both SPD and GLY showed mechanical and barrier characteristics comparable with those of Viscofan NDX [21], a widely commercialized, collagen-derived edible film largely used in casings for fresh or processed sausages, or dry-cured snacks. Therefore, plant-derived casings made with BVPC and low amounts of both SPD and GLY deserve to be produced and tested as possible alternatives to the casings of animal origin.

## 3. Materials and Methods

### 3.1. Materials

Bitter vetch seeds were obtained from a local market in Gallicchio (PZ), Italy. Viscofan NDX edible casings were from Naturin Viscofan GmbH (Tajonar-Navarra, Spain); Mater-Bi (S 301) and high density polyethylene (HD-PET) materials were from local market shopping bags, Naples, Italy. SPD was from Sigma Chemical Company (St. Louis, MO, USA), GLY (about 87%) was from the Merck Chemical Company (Darmstadt, Germany), and all other chemicals and solvents used in this study were analytical grade commercial products.

### 3.2. BVPC Film Preparation

BVPC, the derived FFSs, and films containing or not different concentrations of SPD and, or GLY, were prepared as previously described [7]. The flour obtained from BV seeds grinded in a rotary mill (Grindomix GM200, Retsch GmbH, Haan, Germany) at a speed of 1300 r.p.m. for 5 min was dispersed in distilled water (1:10, *w*/*v*), brought to pH 11.0 with 0.1 N NaOH, and stirred at medium speed for 1 h at room temperature. The suspension was centrifuged at 3200× *g* for 10 min and the pH of the collected supernatant was adjusted to 5.4 by 0.1 N HCl addition. The obtained precipitate was then separated by a new centrifugation at 3200× *g* for 10 min, poured, uniformly distributed on a plastic plate, and dried at 37 °C and 25% relative humidity (RH). The obtained BVPC was finally grinded and dispersed in distilled water (2 g/100 mL), and the pH value was adjusted to pH 12.0 by using 0.1 N NaOH under constant stirring until the powder was completely solubilized. Aliquots of BVPC solution were brought to pH 8.0 and 11.0, respectively, by 0.1 N HCl and then incubated in the presence of different concentrations of SPD (2–5 mM, corresponding to about 4–9% *w*/*w* BV protein) for 30 min, either at 25 or 80 °C to obtain FFSs containing both native and denatured BV protein samples. Where indicated, increasing concentrations of GLY (4–42 mM GLY, corresponding to about 5–50% *w*/*w* BV protein) were added to the obtained FFSs at the end of incubation. All the different FFSs (50 mL), containing or not SPD and, or GLY, were poured onto 8-cm-diameter polystyrene Petri dishes (7.8 mg protein/cm^2^) and allowed to dry in an environmental chamber at 25 °C and 45% RH for 48 h. Finally, the dried films were peeled from the casting surface and stored at 25 °C and 50% RH. Film sample strips (10 mm wide and 50 mm long), obtained by using a sharp razor blade, were conditioned in an environmental chamber at 25 °C and 50% RH for 2 h by placing them into a dessicator over a saturated solution of Mg(NO_3_)_2_·6H_2_O before being tested.

### 3.3. BVPC Film Properties

Film thickness was measured in six different points with a micrometer (Electronic digital micrometer, DC-516, sensitivity 0.001 mm) and film TS at break, EB, and YM were determined in five specimens for each sample (1 KN load and 1 mm/5 min speed) as previously reported [26], by using an Instron universal testing instrument model No. 5543A (Instron Engineering Corp., Norwood, MA, USA). 

The measurements of film permeability to O_2_ [27], CO_2_ [28] and water vapor (WV) [29] were determined in triplicate for each sample at 25 °C under 50% RH by using a TotalPerm apparatus (ExtraSolution s.r.l., Pisa, Italy). 

Film surface and cross section morphology were observed using a Scanning Electron Microscope (Nova NanoSem 450-FEI) (SEM). For film surface analysis, the samples were placed on an aluminum stub by using a graphite double-sided adhesive tape, whereas for film cross sections the samples were fractured in liquid nitrogen and rested vertically on the sides of a rectangular aluminum piece fixed on stubs using a double-sided adhesive tape. A thin coat of gold and palladium was sputtered at a current of 20 mA for 120 s. The sputter-coated samples were then introduced into the specimen chamber and the images were acquired at an accelerating voltage of 3 kV, through the Everhart Thornley Detector. Micrographs for sample surfaces and cross sections were obtained at 7000× magnifications.

### 3.4. Statistical Analysis

JMP software 10.0 (SAS Institute, Cary, NC, USA) was used for all statistical analyses. The data were subjected to the analysis of variance (*F*-test) to evaluate the effect of SPD and GLY on film mechanical and barrier properties (significance at *p* < 0.05), whereas the means of the obtained data were analyzed using the Tukey-Kramer HSD (*t-*test) for pair comparison between control and treated samples (significance at *p* < 0.05).

## 4. Conclusions

Our findings suggest that the use of SPD or of a combination of the polyamine with a primary plasticizer such as GLY as additives of protein-based films, may open new possibilities to generate hydrocolloid edible films endowed with different mechanical and barrier properties specifically suitable for the coating of different food products. In fact, one of the main technical challenges in food processing and storage today is the development of tailor-made coating materials with appropriate characteristics according to the specific requirements of the various fresh or processed foodstuffs: meat, fish, dairy products, fruit, vegetables, as well as ready-to-eat meals [30]. In fact, although desirable mechanical and permeability properties remain the main factors to consider when selecting packaging materials, these features are particularly strategical for any edible coating in the processing and end use of food products, representing the main parameters to ensure food integrity against mechanical damage, microbial spoilage, and duration of the guarantee term. 

## Figures and Tables

**Table 1:** Effect of different concentrations of spermidine (SPD) and glycerol (GLY) on the tensile strength (TS) (MPa) of native (A) and heat-denatured (B) bitter vetch (BV) protein films obtained at pH 8.0 *.

Addition		GLY, mM						
		0	4	8	17	25	33	42
A	none	ND	ND	ND	ND	ND	1.42 ± 0.72	1.60 ± 0.40
	+2 mM SPD	ND	ND	ND	ND	1.28 ± 0.65	1.02 ± 0.26 ^a^	1.42 ± 0.07 ^a^
	+3 mM SPD	ND	3.49 ± 0.40	1.33 ± 0.24	1.47 ± 0.14	1.03 ± 0.42	0.58 ± 0.07 ^a^	0.39 ± 0.05 ^a^
	+4 mM SPD	3.45 ± 0.41	8.38 ± 1.90 ^b^	6.24 ± 0.80 ^b^	2.03 ± 0.20 ^b^	1.32 ± 0.32 ^b^	0.61 ± 0.04 ^a,b^	0.58 ± 0.02 ^a,b^
	+5 mM SPD	3.03 ± 1.61	3.44 ± 1.58	5.30 ± 1.33	2.91 ± 0.36	1.66 ± 0.43 ^b^	0.62 ± 0.05 ^a,b^	0.66 ± 0.08 ^a,b^
B	none	ND1	ND1	ND1	ND1	ND1	ND1	ND1
	+2 mM SPD	7.14 ± 0.24	7.78 ± 1.04	4.43 ± 0.80 ^b^	2.34 ± 0.40 ^b^	2.34 ± 0.40 ^b^	1.18 ± 0.34 ^b^	ND1
	+3 mM SPD	12.00 ± 1.21	7.65 ± 0.67 ^b^	4.95 ± 1.24 ^b^	2.66 ± 0.95 ^b^	1.76 ± 0.72 ^b^	1.42 ± 0.24 ^b^	1.17 ± 0.42 ^b^
	+4 mM SPD	7.96 ± 1.33	6.72 ± 1.96	6.79 ± 2.15	3.08 ± 1.39 ^b^	1.60 ± 0.36 ^b^	0.89 ± 0.30 ^b^	0.95 ± 0.38 ^b^
	+5 mM SPD	5.71 ± 1.76	4.02 ± 1.33	3.23 ± 0.70	1.25 ± 0.58 ^b^	1.05 ± 0.22 ^b^	0.96 ± 0.11 ^b^	0.86 ± 0.40 ^b^

* Not detectable values because of the film brittleness (ND) or stickiness (ND1). Significantly different values as compared to the ones obtained under the same experimental conditions without SPD (^a^) or GLY (^b^) (*t*-test, *p* < 0.05); *F*-test was positive (*p* < 0.05) for variations of TS with SPD and GLY concentrations. Further experimental details are given in the text.

**Table 2:** Effect of different concentrations of SPD and GLY on the elongation at break (EB, %) of native (A) and heat- denatured (B) BV protein films obtained at pH 8.0 *.

Addition		GLY, mM						
		0	4	8	17	25	33	42
A	none	ND	ND	ND	ND	ND	15.21 ± 2.18	35.08 ± 3.43
	+2 mM SPD	ND	ND	ND	ND	40.61 ± 2.41	54.69 ± 5.45 ^a^	60.16 ± 4.52 ^a^
	+3 mM SPD	ND	24.62 ± 4.79	47.94 ± 4.56	50.28 ± 2.85	59.36 ± 6.25	64.41 ± 4.12 ^a^	72.31 ± 8.34 ^a^
	+4 mM SPD	0.89 ± 0.05	8.44 ± 2.67 ^b^	31.15 ± 3.85 ^b^	55.82 ± 4.67 ^b^	64.55 ± 5.48 ^b^	67.40 ± 6.64 ^a,b^	71.78 ± 2.05 ^a,b^
	+5 mM SPD	1.05 ± 0.15	4.56 ± 0.87 ^b^	9.28 ± 4.38 ^b^	22.78 ± 3.69 ^b^	60.23 ± 7.20 ^b^	72.58 ± 5.10 ^a,b^	86.26 ± 3.04 ^a,b^
B	none	ND1	ND1	ND1	ND1	ND1	ND1	ND1
	+2 mM SPD	1.00 ± 0.01	5.35 ± 1.73 ^b^	13.11 ± 3.25 ^b^	32.91 ± 3.34 ^b^	35.85 ± 1.59 ^b^	59.02 ± 4.48 ^b^	ND1
	+3 mM SPD	3.80 ± 0.80	15.17 ± 2.19 ^b^	24.66 ± 2.81 ^b^	33.26 ± 4.93 ^b^	37.02 ± 2.84 ^b^	51.57 ± 4.56 ^b^	80.88 ± 4.92 ^b^
	+4 mM SPD	6.44 ± 1.54	14.60 ± 2.48 ^b^	25.50 ± 2.59 ^b^	34.64 ± 3.24 ^b^	40.66 ± 5.74 ^b^	42.05 ± 3.68 ^b^	44.52 ± 4.89 ^b^
	+5 mM SPD	9.94 ± 1.66	18.01 ± 3.52 ^b^	32.38 ± 3.44 ^b^	34.51 ± 4.76 ^b^	46.66 ± 4.58 ^b^	20.36 ± 3.21 ^b^	21.45 ± 2.71 ^b^

* Not detectable values because of the film brittleness (ND) or stickiness (ND1). Significantly different values as compared to the ones obtained under the same experimental conditions without SPD (^a^) or GLY (^b^) (*t*-test, *p* < 0.05); *F*-test was positive (*p* < 0.05) for variations of EB with SPD and GLY concentrations. Further experimental details are given in the text.

**Table 3:** Effect of different concentrations of SPD and GLY on the Young’s module (YM, MPa) of native (A) and heat-denatured (B) BV protein films obtained at pH 8.0 *.

Addition		GLY, mM						
		0	4	8	17	25	33	42
A	none	ND	ND	ND	ND	ND	80.0 ± 1.4	30.7 ± 0.6
	+2 mM SPD	ND	ND	ND	ND	25.4 ± 1.6	18.4 ± 3.6 ^a^	9.7 ± 1.5 ^a^
	+3 mM SPD	ND	154.9 ± 16.0	125.2 ± 13.6	23.8 ± 2.5	12.4 ± 2.4	14.3 ± 2.3 ^a^	4.3 ± 0.5 ^a^
	+4 mM SPD	425.3 ± 25.8	381.9 ± 17.1	121.9 ± 12.4 ^b^	42.2 ± 5.6 ^b^	16.1 ± 3.3 ^b^	13.1 ± 3.8 ^a,b^	3.5 ± 0.2 ^a.b^
	+5 mM SPD	397.8 ± 20.0	320.1 ± 27.1 ^b^	185.7 ± 19.3 ^b^	59.8 ± 4.0 ^b^	10.0 ± 1.3 ^b^	6.9 ± 0.5 ^a,b^	2.9 ± 0.5 ^a.b^
B	none	ND1	ND1	ND1	ND1	ND1	ND1	ND1
	+2 mM SPD	804.8 ± 50.5	325.0 ± 17.0 ^b^	184.5 ± 4.3 ^b^	63.1 ± 4.5 ^b^	26.3 ± 4.2 ^b^	11.8 ± 1.1 ^b^	ND1
	+3 mM SPD	454.0 ± 54.2	310.5 ± 25.1 ^b^	143.5 ± 5.4 ^b^	48.0 ± 1.6 ^b^	23.7 ± 4.0 ^b^	22.1 ± 3.8 ^b^	7.1 ± 1.3 ^b^
	+4 mM SPD	356.7 ± 20.5	196.4 ± 15.5 ^b^	150.2 ± 9.5 ^b^	46.5 ± 6.5 ^b^	22.5 ± 3.5 ^b^	21.9 ± 5.0 ^b^	17.7 ± 5.3 ^b^
	+5 mM SPD	226.6 ± 6.6	80.7 ± 3.7 ^b^	82.1 ± 3.4 ^b^	38.4 ± 4.2 ^b^	21.7 ± 2.1 ^b^	23.3 ± 6.0 ^b^	16.1 ± 5.8 ^b^

* Not detectable values because of the film brittleness (ND) or stickiness (ND1). Significantly different values as compared to the ones obtained under the same experimental conditions without SPD (^a^) or GLY (^b^) (*t*-test, *p* < 0.05); *F*-test was positive (*p* < 0.05) for variations of YM with SPD and GLY concentrations. Further experimental details are given in the text.

**Table 4:** Effect of different GLY and SPD concentrations on gas permeability (cm^3^ mm m^−2^ day^-1^ kPa^−1^) of native (A) and heat-denatured (B) BV protein films obtained at pH 8.0 *.

Addition		CO_2_ Permeability				O_2_ Permeability			
		GLY, mM							
		0	8	25	42	0	8	25	42
A	none	ND	ND	ND	3.025 ± 0.210	ND	ND	ND	1.910 ± 0.013
	+3 mM SPD	ND	4.520 ± 0.685	0.990 ± 0.264	1.750 ± 0.163 ^a^	ND	8.107 ± 0.579	0.037 ± 0.010	0.059 ± 0.008 ^a^
	+4 mM SPD	7.592 ± 0.597	0.236 ± 0.062 ^b^	0.538 ± 0.084 ^b^	1.768 ± 0.313 ^a,b^	1.780 ± 0.104	0.017 ± 0.002 ^b^	0.048 ± 0.001 ^b^	0.078 ± 0.009 ^a,b^
	+5 mM SPD	0.982 ± 0.208	1.240 ± 0.140	2.613 ± 0.559 ^b^	2.396 ± 0.259 ^a,b^	0.293 ± 0.091	0.011 ± 0.008 ^b^	0.086 ± 0.006 ^b^	0.063 ± 0.001 ^a,b^
B	none	ND1	ND1	ND1	ND1	ND1	ND1	ND1	ND1
	+3 mM SPD	15.910 ± 2.030	0.380 ± 0.031 ^b^	0.567 ± 0.040 ^b^	0.630 ± 0.020 ^b^	3.559 ± 0.421	0.018 ± 0.002 ^b^	0.047 ± 0.003 ^b^	0.065 ± 0.004 ^b^
	+4 mM SPD	1.250 ± 0.241	0.463 ± 0.093 ^b^	0.389 ± 0.012 ^b^	2.321 ± 0.540 ^b^	2.130 ± 0.240	0.026 ± 0.004 ^b^	0.042 ± 0.001 ^b^	0.057 ± 0.003 ^b^
	+5 mM SPD	0.322 ± 0.007	0.664 ± 0.035 ^b^	0.823 ± 0.064 ^b^	2.319 ± 0.106 ^b^	1.671 ± 0.113	0.031 ± 0.002 ^b^	0.052 ± 0.012 ^b^	0.090 ± 0.001 ^b^

* Not detectable values because of film brittleness (ND) or stickiness (ND1). Significantly different values as compared to the ones obtained under the same experimental conditions without SPD (^a^) or GLY (^b^) (*t*-test, *p* < 0.05); *F*-test was positive (*p* < 0.05) for variations of permeability with SPD and GLY concentrations. Further experimental details are given in the text.

**Table 5:** Effect of different GLY and SPD concentrations on water vapor (WV) permeability (cm^3^ mm m^−2^ day^−1^ kPa^−1^) of native (A) and heat-denatured (B) BV protein films obtained at pH 8.0 *.

Addition		WV Permeability			
		GLY, mM			
		0	8	25	42
A	none	ND	ND	ND	0.045 ± 0.007
	+3 mM SPD	ND	0.023 ± 0.001	0.102 ± 0.030	0.240 ± 0.019 ^a^
	+4 mM SPD	0.418 ± 0.010	0.039 ± 0.003 ^b^	0.116 ± 0.014 ^b^	0.228 ± 0.007 ^a,b^
	+5 mM SPD	0.308 ± 0.162	0.073 ± 0.002 ^b^	0.190 ± 0.061 ^b^	0.193 ± 0.001 ^a,b^
B	none	ND1	ND1	ND1	ND1
	+3 mM SPD	0.550 ± 0.045	0.022 ± 0.002 ^b^	0.067 ± 0.003 ^b^	0.088 ± 0.001 ^b^
	+4 mM SPD	0.367 ± 0.042	0.050 ± 0.002 ^b^	0.085 ± 0.001 ^b^	0.169 ± 0.008 ^b^
	+5 mM SPD	0.028 ± 0.004	0.036 ± 0.004	0.070 ± 0.001 ^b^	0.237 ± 0.001 ^b^

* Not detectable values because of film brittleness (ND) or stickiness (ND1). Significantly different values as compared to the ones obtained under the same experimental conditions without SPD (^a^) or GLY (^b^) (*t*-test, *p* < 0.05); *F*-test was positive (*p* < 0.05) for variations of permeability with SPD and GLY concentrations. Further experimental details are given in the text.

**Table 6:** Effect of 5 mM SPD and, or 42 mM GLY on the thickness of native (A) and heat-denatured (B) BV protein films obtained at pH 8.0 *.

	Addition	Thickness, μm
**A**	+GLY	107.93 ± 1.84 ^b^
	+SPD	82.60 ± 1.08 ^a,b^
	+GLY + SPD	100.40 ± 1.76 ^a,b^
**B**	+GLY	ND
	+SPD	76.62 ± 1.01 ^b^
	+GLY + SPD	87.23 ± 1.13 ^b^

* Not detectable value because of film stickiness (ND). The values obtained in the presence of SPD (panel A) were significantly different from those obtained under the same experimental conditions in its absence (^a^) (*t*-test, *p* < 0.05); *F*-test was positive (*p* < 0.05) for variations of thickness with SPD and GLY (^b^). Further experimental details are given in the text.

**Table 7:** Thickness, mechanical, and barrier properties of some commercial materials and of different protein- and polysaccharide-based films described in the literature.

Film	[Reference]	Thickness (µm)	TS(MPa)	EB(%)	YM (MPa)	Permeability (cm^3^ mm m^−2^ day^−1^ kpa^−1^)		
						CO_2_	O_2_	WV
**Protein-Based ^a^**								
SP + 30% GLY	[22]	98 ± 7	3.8 ± 0.4	6.5 ± 0.6	ND	ND	0.0012	0.43 ± 0.04
SP + 40% SOR	[22]	106 ± 7	4.0 ± 0.2	7.2 ± 1.1	ND	ND	0.0011	0.02 ± 0.01
WG + 20% GLY	[23]	53	2.6 ± 1.1	22.0 ± 10.0	2.6 ± 1.1	ND	ND	0.54
WP + 40% GLY	[24]	120 ± 8	0.83 ± 0.01	51.0 ± 1.0	43.0 ± 1.5	1.02 ± 0.01	0.20	8.25 ± 0.31
**Polysaccharide-Based ^b^**								
CHI + 25% GLY	[25]	55 ± 1	15.1 ± 0.5	22.1 ± 2.1	ND	0.02 ± 0.01	0.025	0.0005
PEC + 30% GLY	[6]	90 ± 1.2	11.0 ± 1.0	6.9 ± 0.2	ND	0.02 ± 0.01	ND	9.8 ± 0.4
PEC + 8 mM SPD	[6]	70 ± 0.5	5.6 ± 0.3	8.3 ± 0.6	ND	0.08 ± 0.01	ND	20.3 ± 0.3
PEC + GLY + SPD	[6]	101 ± 1.3	2.7 ± 0.3	28.1 ± 1.2	ND	1.02 ± 0.03	ND	10.2 ± 0.2
**Commercial ^c^**								
Viscofan (NDX)	[PS]	30.0 ± 0.4	36.6 ± 8.1	13.1 ± 2.9	356 ± 29	3.71 ± 0.16	0.03 ± 0.01	0.08 ± 0.01
Mater-Bi (S-301)	[PS]	16.0 ± 1.3	18.4 ± 2.7	317.9 ± 35.9	42.9 ± 0.8	5.23 ± 0.01	0.74 ± 0.01	0.04 ± 0.01
HD-PE 02	[PS]	36.2 ± 1.7	13.1 ± 1.4	501.9 ± 43.3	75.2 ± 2.7	10.89 ± 1.48	3.21 ± 0.59	0.0002

^a^ SP, soy protein; SOR, sorbitol; WG, wheat gluten; WP, whey protein. ^b^ CHI, chitosan; PEC, pectin; ND, not detected. ^c^ The results were obtained in the present study [PS] and are expressed as means ± standard deviation, either of six specimens for the determination of thickness and mechanical properties or of three specimens analyzed in permeability tests. Further experimental details are described under Materials and Methods.
